# Inflammatory pseudotumor-like follicular dendritic cell sarcoma of the spleen: A diagnostic challenge with imaging–pathologic correlation

**DOI:** 10.1016/j.radcr.2026.06.075

**Published:** 2026-07-04

**Authors:** Liying Cheng, ShengYe Wu, JiBo Hu

**Affiliations:** Department of Radiology, the Fourth Affiliated Hospital of School of Medicine and International School of Medicine, International Institutes of Medicine, Zhejiang University, Yiwu 322000, China

**Keywords:** Epstein–Barr virus (EBV), Follicular dendritic cell sarcoma (FDCS), Inflammatory pseudotumor-like, Spleen, Case report, Imaging–pathologic correlation

## Abstract

Epstein–Barr virus (EBV)-associated inflammatory pseudotumor-like follicular dendritic cell sarcoma (IPT-like FDCS) is a rare neoplasm that most commonly arises in the spleen or liver and is frequently misdiagnosed due to nonspecific clinical and imaging features. We report the case of a 46-year-old man presenting with intermittent left upper abdominal discomfort and an incidentally detected splenic mass. Ultrasonography and contrast-enhanced computed tomography demonstrated a heterogeneous lesion with relatively marked arterial-phase enhancement and persistent internal heterogeneity on the venous phase. These findings were indeterminate, and a malignant primary splenic neoplasm could not be excluded; laparoscopic splenectomy was therefore performed.

Histopathological examination revealed spindle-to-oval tumor cells within a prominent inflammatory background. Immunohistochemical analysis showed positivity for follicular dendritic cell markers, including CD21 and CD23, and in situ hybridization confirmed Epstein–Barr virus-encoded RNA (EBER) positivity, establishing the diagnosis of IPT-like FDCS. The postoperative course was uneventful, though no follow-up data were available.

This case highlights the diagnostic difficulty of splenic IPT-like FDCS, which may mimic other benign and malignant splenic tumors on imaging. Imaging heterogeneity in this case directly reflected the tumor's mixed microscopic composition of fibrotic stroma, inflammatory infiltrate, and viable neoplastic tissue. CT findings alone are insufficient for a definitive diagnosis; however, careful assessment of lesion heterogeneity, enhancement pattern, and the absence of overt extra-splenic disease may help include IPT-like FDCS in the differential diagnosis. Correlation with histopathological findings, follicular dendritic cell marker expression, and EBER in situ hybridization is essential for accurate diagnosis.

## Introduction

Inflammatory pseudotumor-like follicular dendritic cell sarcoma (IPT-like FDCS) is a rare and clinicopathologically distinct variant of follicular dendritic cell sarcoma that is strongly associated with Epstein–Barr virus (EBV) infection and most commonly involves the spleen and liver [[1], [2], [3]]. Its prominent inflammatory background may obscure the underlying neoplastic follicular dendritic cell proliferation, contributing to diagnostic difficulty.

Splenic IPT-like FDCS often lacks specific clinical, laboratory, and radiologic features, making preoperative diagnosis challenging. Because its imaging appearance may overlap with a variety of benign and malignant splenic lesions, systematic imaging–pathologic correlation is critical for accurate characterization of this entity [4].

Although reports of IPT-like FDCS have increased, systematic imaging–pathologic correlation remains limited. Herein, we report a case of EBV-associated IPT-like FDCS of the spleen presenting as a solitary heterogeneous mass with relatively marked arterial-phase enhancement, and we discuss its imaging–pathologic correlation and diagnostic pitfalls.

## Case presentation

A 46-year-old man presented to our institution for evaluation of a splenic mass that had been incidentally detected 10 months earlier. At the time of initial detection, he reported mild, intermittent left upper quadrant discomfort that resolved spontaneously within approximately one hour, without associated gastrointestinal or systemic symptoms.

His medical history included hypertension, hyperuricemia, and previous repair of a right inguinal hernia. Physical examination on admission was unremarkable, with no abdominal tenderness or palpable mass. Laboratory tests showed mild elevations in several inflammatory markers, including fibrinogen, serum amyloid A, and high-sensitivity C-reactive protein. Liver enzyme levels, albumin, and creatine kinase were slightly below the reference ranges, and tumor marker levels were normal.

Initial ultrasonography showed a hypoechoic mass in the spleen with an irregular but relatively well-circumscribed margin and scant internal vascularity on color Doppler imaging (Fig. 1). On unenhanced CT, the lesion appeared heterogeneous and hypoattenuating (Fig. 2); unlike its sonographic appearance, lesion margins were indistinct from the adjacent splenic parenchyma, likely owing to the limited attenuation difference between the tumor and surrounding parenchyma on unenhanced images.On contrast-enhanced CT, the mass showed avid but heterogeneous enhancement in the arterial phase, with focal hypoenhancing areas (Fig. 3).Enhancement decreased mildly in the venous phase but remained heterogeneous (Fig. 4).

**Fig. 1:**
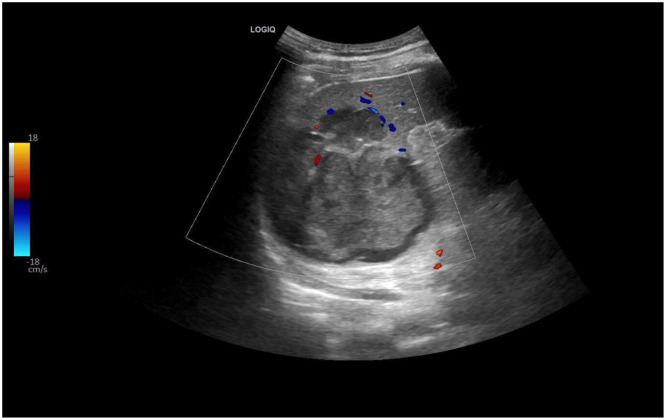
Ultrasonography demonstrating an irregular, relatively well-circumscribed hypoechoic splenic mass with scant internal vascularity on color Doppler imaging.

**Fig. 2:**
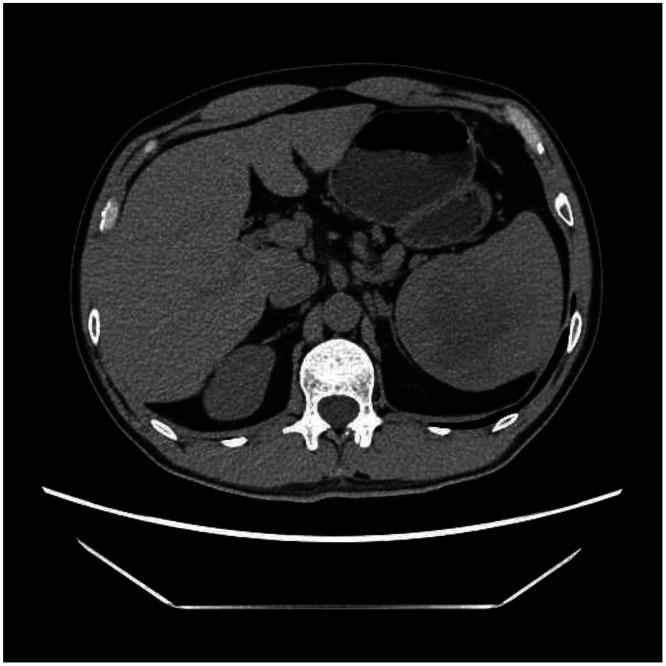
Unenhanced CT revealing a heterogeneous, hypoattenuating splenic lesion with indistinct margins relative to the surrounding parenchyma.

**Fig. 3:**
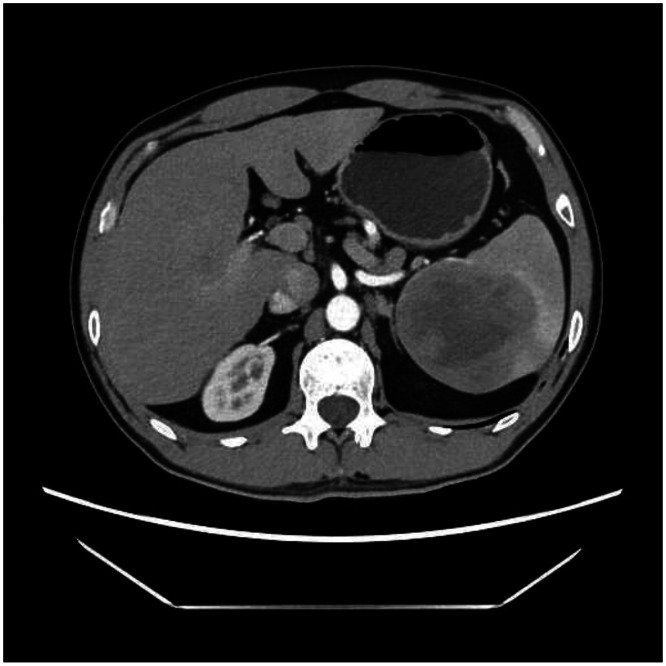
Contrast-enhanced CT showing avid heterogeneous arterial-phase enhancement with focal hypoenhancing areas.

**Fig. 4:**
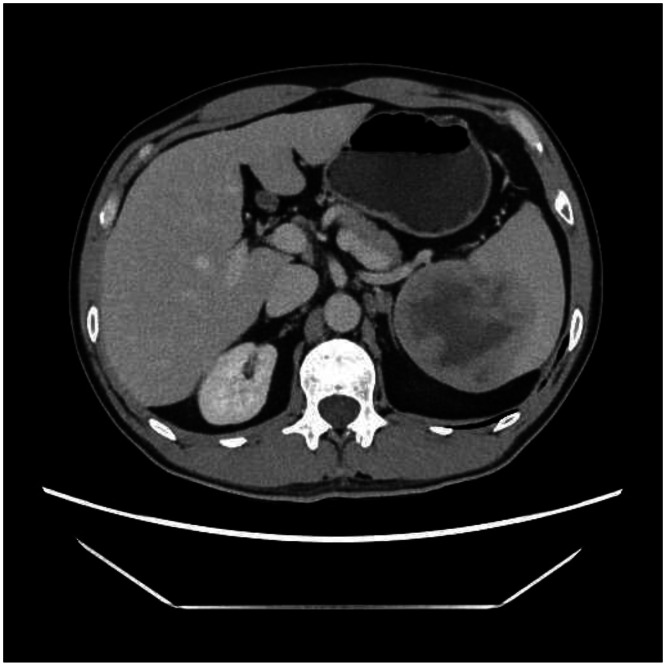
Contrast-enhanced CT in the portal venous phase showing persistent heterogeneous enhancement with residual hypoenhancing areas.

Because the imaging findings were indeterminate and primary splenic malignancy could not be excluded, laparoscopic splenectomy was performed. The resected spleen measured approximately 15 × 7 cm. Histological examination showed loosely arranged spindle-to-oval tumor cells within a fibrous stroma, accompanied by a prominent inflammatory infiltrate composed mainly of lymphocytes and plasma cells (Fig. 5). Immunohistochemistry demonstrated positive expression of the follicular dendritic cell markers CD21 and CD23 in the tumor cells (Fig. 6). CD20 staining was negative in the tumor cells while showing strong positivity in the surrounding background lymphocytes, confirming their B-cell nature and highlighting the neoplastic population by contrast (Fig. 7). In situ hybridization for Epstein–Barr virus–encoded RNA showed diffuse nuclear positivity in the tumor cells (Fig. 8).

**Fig. 5:**
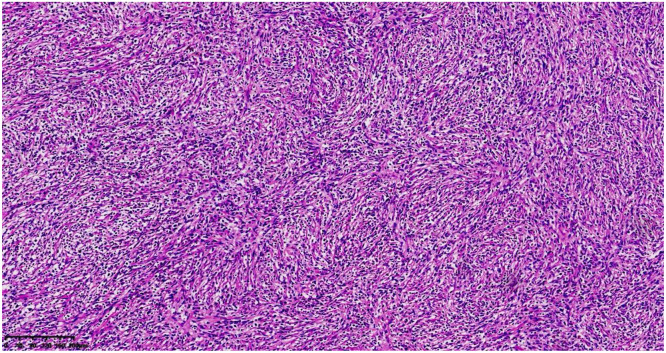
Histological examination showing loosely arranged spindle-to-oval tumor cells in a fibrous stroma with a prominent lymphoplasmacytic infiltrate.

**Fig. 6:**
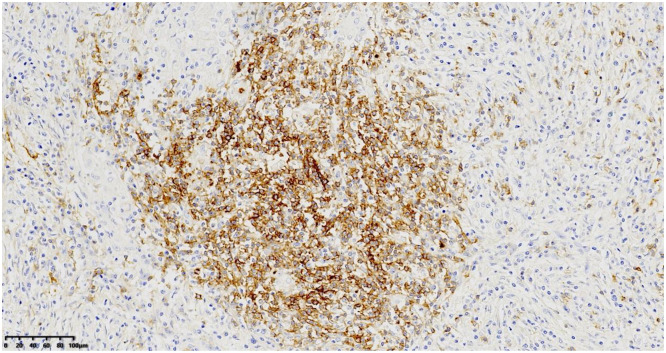
CD21 immunohistochemistry of the tumor. Strong CD21 positivity highlights the larger tumor cells with a characteristic, dense positive meshwork pattern, delineating the complex dendritic cell processes.

**Fig. 7:**
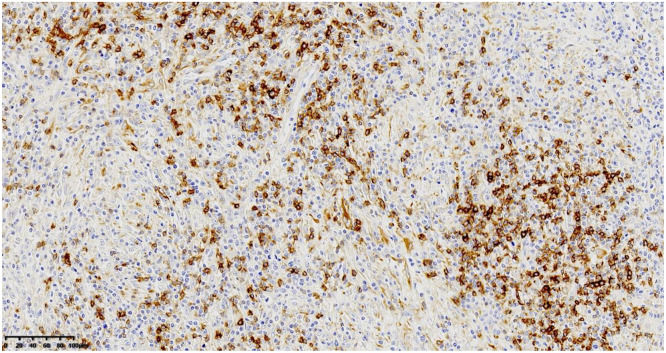
Dual CD20/CD23 immunohistochemistry of the tumor. Cocktail staining demonstrates strong CD20 positivity in the numerous small round background B lymphocytes, with concurrent CD23 expression in the larger neoplastic cells, enabling clear delineation of the two populations.

**Fig. 8:**
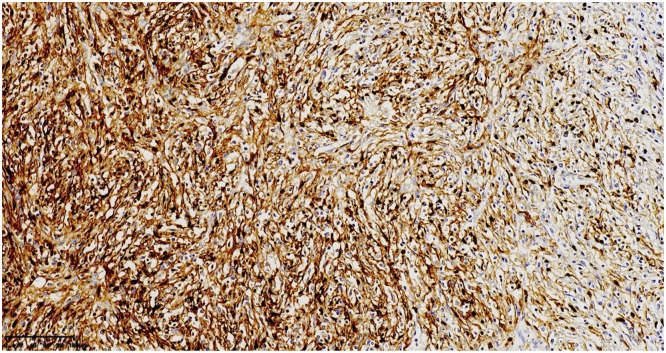
In situ hybridization showing diffuse nuclear positivity for Epstein-Barr virus-encoded RNA (EBER) in the tumor cells.

## Discussion

Follicular dendritic cell sarcoma (FDCS) is a rare neoplasm derived from follicular dendritic cells, which are mesenchymal-derived stromal cells of lymphoid follicles [5]. The inflammatory pseudotumor-like variant (IPT-like FDCS) is a recognized clinicopathological subtype that preferentially involves the liver and spleen, set apart from conventional FDCS by its striking inflammatory stromal infiltrate and consistent association with Epstein–Barr virus (EBV) [2]. In cases where disease remains confined to the spleen and complete resection is achieved, the clinical course tends to be indolent; nonetheless, recurrence and distant metastasis have been documented in a small proportion of patients [6]; Despite its comparatively favorable behavior, the rarity of IPT-like FDCS and the absence of specific clinical features continue to make preoperative diagnosis difficult. A high index of clinical suspicion is therefore required.

The clinical presentation of splenic IPT-like FDCS is largely nonspecific. Most patients are either asymptomatic or report only vague upper abdominal discomfort. Frank abdominal pain, constitutional symptoms, and tumor marker elevation are characteristically absent [7]. In the present case, the patient described mild, intermittent left upper quadrant discomfort, and a comprehensive tumor marker panel was unremarkable. Mildly elevated fibrinogen, serum amyloid A, and high-sensitivity C-reactive protein were noted and may reflect the underlying inflammatory environment of the tumor, though such findings are neither sensitive nor specific enough to guide diagnosis. The absence of distinctive clinical or laboratory features not only complicates early recognition but also increases the risk of preoperative misclassification. In practice, this diagnosis is rarely considered before surgery.

Imaging is central to the detection and initial characterization of splenic lesions, yet the imaging manifestations of splenic IPT-like FDCS are variable and lack pathognomonic features [8]. On ultrasonography, smaller lesions typically appear as well-circumscribed hypoechoic masses, whereas larger lesions often exhibit heterogeneous internal echogenicity, occasionally with branching or linear hyperechoic components. Color Doppler imaging may demonstrate absent or markedly diminished internal vascularity, with only sparse linear vascular signals identified in some cases [9]. On CT, splenic IPT-like FDCS most commonly manifests as a solitary, round-to-oval, iso- to hypodense mass on unenhanced images, with lesion margins becoming more conspicuous following contrast administration. Contrast-enhanced CT typically demonstrates mild-to-moderate persistent enhancement, with the lesion remaining hypovascular relative to the surrounding splenic parenchyma, with delayed peripheral rim or capsular enhancement observed in a subset of cases. Larger lesions may exhibit heterogeneous attenuation resulting from internal necrosis, cystic degeneration, or dystrophic calcification [10]. On MRI, variable signal intensities (e.g., T1 hyperintensity, T2 hypointensity) have been reported, while ¹⁸F-FDG PET/CT may demonstrate increased tracer uptake, further reflecting the imaging variability of this entity [11]. No single imaging feature is specific for IPT-like FDCS.

On reviewing the histological sections alongside the CT images, we noted a close correspondence between the tumor's microscopic composition and its imaging features. The lesion consisted of spindle-to-oval cells within a fibrous stroma, interspersed with dense lymphocytic and plasmacytic infiltrates and focal degenerative areas. This mixed composition directly explains the imaging heterogeneity: hypoenhancing foci on contrast-enhanced CT corresponded to fibrotic or degenerative zones with reduced cellularity, while the more avidly enhancing regions reflected viable neoplastic tissue supported by a vascularized inflammatory stroma. Rather than representing a specific diagnostic criterion, imaging heterogeneity in splenic IPT-like FDCS is better understood as a direct reflection of the tumor's complex internal architecture. Tissue sampling therefore remains indispensable.

Several imaging features of this case deserve further comment in the context of the broader spectrum described in the literature. The ill-defined margins on unenhanced CT should not rule out a diagnosis of splenic IPT-like FDCS, as prior reports have demonstrated that lesion margins may become more conspicuous only following contrast administration. Although the degree of arterial enhancement in our case was more pronounced than the mild-to-moderate persistent enhancement described in most previously reported cases, such variability likely reflects differences in intratumoral vascularity, the extent of inflammatory stroma, fibrous composition, and the degree of internal necrosis or cystic degeneration [2,12]. Marked heterogeneous enhancement should therefore not be considered incompatible with a diagnosis of splenic IPT-like FDCS. Rather than yielding a definitive preoperative diagnosis, imaging serves primarily to identify an indeterminate splenic mass, characterize its internal architecture and enhancement pattern, and guide subsequent histopathological evaluation. Imaging alone cannot establish this diagnosis.

The differential diagnosis of splenic IPT-like FDCS is broad, including lymphoma, metastatic disease, inflammatory pseudotumor, and other primary splenic spindle-cell neoplasms. Splenic lymphoma may manifest as solitary or multifocal hypodense lesions with mild enhancement, and the presence of associated lymphadenopathy, splenomegaly, or systemic symptoms may favor this diagnosis. In the present case, the absence of lymphadenopathy, splenomegaly, and systemic symptoms argued against lymphoma. Splenic metastases may similarly appear as hypoenhancing nodules [13,14]; however, they are more frequently multifocal and typically arise in the setting of a known primary malignancy. Inflammatory pseudotumor represents a particularly challenging diagnostic mimic as it may share overlapping radiologic and histopathological features with IPT-like FDCS. However [7], IPT lacks neoplastic FDC proliferation and is consistently negative for FDC markers and EBER, in contrast to IPT-like FDCS, enabling definitive distinction through integrated immunohistochemical and molecular analysis [7]. Other primary splenic spindle-cell neoplasms, including splenic sarcomas, may demonstrate irregular enhancement and malignant spindle-cell proliferation; however, they characteristically lack the combination of a dense inflammatory background, follicular dendritic cell immunophenotype, and EBV association that is characteristic of IPT-like FDCS. Definitive distinction among these entities requires integrated histopathological examination, immunohistochemistry, and EBER in situ hybridization, and cannot be established by imaging alone.

The clinical relevance of this case lies in the diagnostic ambiguity arising from mild symptoms, nonspecific inflammatory marker elevation, and heterogeneous imaging findings that overlap with several other splenic lesions. For radiologists, awareness of the variable enhancement spectrum of splenic IPT-like FDCS — including the more pronounced arterial-phase enhancement demonstrated here — may help avoid overconfidence in an imaging-based diagnosis and prompt timely referral for histopathological work-up incorporating follicular dendritic cell markers and EBER in situ hybridization. The primary limitation of this report is the absence of follow-up data, precluding assessment of long-term outcome and recurrence risk in this patient.

## Conclusion

This case reinforces the importance of recognizing Epstein–Barr virus-associated inflammatory pseudotumor-like follicular dendritic cell sarcoma as a rare but distinct splenic neoplasm that frequently presents with nonspecific clinical manifestations and heterogeneous imaging features. Here, the relatively marked arterial-phase enhancement and persistent internal heterogeneity expanded the CT spectrum of splenic IPT-like FDCS but were not pathognomonic. The marked inflammatory background may obscure the underlying neoplastic follicular dendritic cell proliferation, and the expression of FDC markers may be focal and subtle; therefore, a targeted immunohistochemical panel and routine EBER in situ hybridization are essential to avoid misclassification. Given these diagnostic considerations, IPT-like FDCS should be considered in the differential diagnosis of a solitary heterogeneous splenic mass, particularly when lymphoma, metastasis, inflammatory pseudotumor, and other spindle-cell splenic lesions cannot be confidently excluded by imaging alone. Complete surgical excision remains the cornerstone of treatment for localized splenic disease and may provide favorable disease control; however, long-term follow-up remains warranted because recurrence or metastasis has occasionally been reported.

## Ethical approval

This study was conducted in accordance with institutional ethical standards.

## Author contributions

**JiBo Hu:** Supervision, conceptualization, final approval. **Liying Cheng:** Writing – original draft. **ShengYe Wu:** Writing – review and editing.

## Patient consent

Written informed consent for publication of this case and accompanying images was obtained from the patient.
